# Crystal structure of 4-[(2-hy­droxy-3-meth­oxy­benz­yl)amino]­benzoic acid hemihydrate

**DOI:** 10.1107/S2056989018018455

**Published:** 2019-01-08

**Authors:** Saima Kamaal, Md. Serajul Haque Faizi, Arif Ali, Musheer Ahmad, Mayank Gupta, Necmi Dege, Turganbay Iskenderov

**Affiliations:** a Department of Applied Chemistry, Faculty of Engineering & Technology, Aligarh Muslim University, Aligarh UP 202 002, India; b Department of Chemistry, Langat Singh College, B. R. A. Bihar University, Muzaffarpur, Bihar 842 001, India; c Department of Chemistry, Indian Institute of Technology Kanpur 208016 UP, India; d Ondokuz Mayıs University, Arts and Sciences Faculty, Department of Physics, 55139 Samsun, Turkey; e National Taras Shevchenko University, Department of Chemistry, Volodymyrska str., 64, 01601 Kyiv, Ukraine

**Keywords:** crystal structure, 2-hy­droxy-3-meth­oxy-benzaldehyde, 4-amino­benzoic acid (PABA), secondary amine, hydrogen bonding, vanillin

## Abstract

In the crystal of the title vanilline derivative, 2C_15_H_15_NO_4_·H_2_O, the secondary amine mol­ecule is accompanied by half equivalent of water. The mol­ecule is non-planar, with torsion angle C_ar­yl_—CH_2_—NH—C_ar­yl_ of −83.9 (2)°. In the crystal, the system of O—H⋯O hydrogen bonds, including bridging water mol­ecules residing on crystallographic twofold axes, results in a two-dimensional layered structure. Within the layers, there are also weak N—H⋯*π* inter­actions involving the vanilline benzene ring.

## Abstract 2

In the crystal, the system of O—H⋯O hydrogen bonds, including bridging water mol­ecules residing on crystallographic twofold axes, results in a two-dimensional layered structure. Within the layers, there are also weak N—H⋯*π* inter­actions involving the vanilline benzene ring.

## Chemical context   

The title compound is obtained by reduction of reported (Kamaal *et al.*, 2018 ▸) (*E*)-4-(2-hy­droxy-3-meth­oxy­benzyl­idene­amino)benzoic acid with sodiumborohydride. The Schiff base is formed by condensation of 4-amino­benzoic acid with *o*-vanilline. Both *p*-amino­benzoic acid and *o*-vanilline have biological importance, for example as a bacterial cofactor involved in the synthesis of folic acid (Robinson, 1966 ▸). Another example is benzocaine, the ethyl ester of *p*-amino­benzoic acid, which is a local anaesthetic. The mechanism includes inhibiting voltage-dependent sodium channels on the nerve membrane, which results in stopping the signal propagation (Neumcke *et al.*, 1981 ▸). The present work is also a part of an ongoing structural study of Schiff bases and secondary amines for their utilization in the synthesis of new organic compounds and application of excited-state proton transfer and fluorescent chemosensor (Faizi *et al.*, 2016*a* ▸,*b* ▸, 2018*a* ▸,*b* ▸; Kumar *et al.*, 2018 ▸; Mukherjee *et al.*, 2018 ▸).
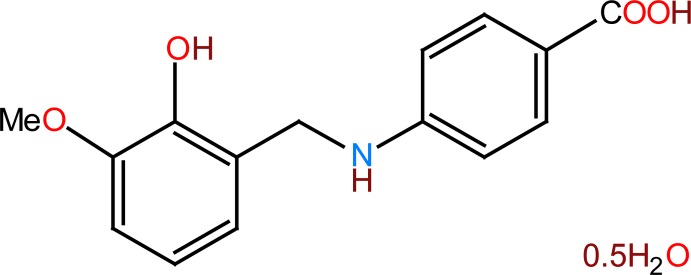


## Structural commentary   

**Figure 1:**
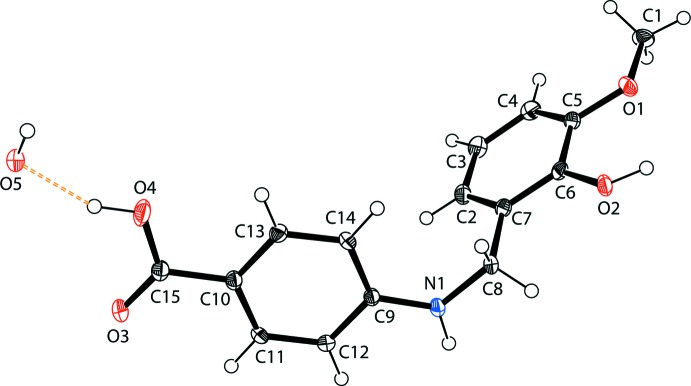
The mol­ecular structure of the title compound, showing the atom labelling. The inter­molecular O—H⋯O hydrogen bond involving the water mol­ecule is shown as a dashed line (see Table 1 ▸ for details). Displacement ellipsoids are drawn at the 40% probability level.

The mol­ecular structure of the title compound is illustrated in Fig. 1 ▸. The title compound has two substituted aromatic rings at either end of the –CH_2_-NH– linkage [C_ar­yl_—CH_2_—NH—C_ar­yl_ torsion angle = −83.9 (2)°]. The water solvent stabilizes the crystal structure through hydrogen bonding. The secondary amine N atom has a practically planar trigonal configuration deviating by just 0.03 (1) Å from the mean plane of the adjacent atoms, and it is apparently conjugated with the adjacent benzene ring [the C—N bond length is 1.368 (2) Å]. For comparison, the reported C—N distance in crystal structure of the ethyl 4-[(*E*)-(4-hy­droxy-3-meth­oxy­benzyl­idene)amino]­benzoate Schiff base is 1.274 (2) Å (Ling *et al.*, 2016 ▸) and in the zwitterion it is 1.312 Å (Kamaal *et al.*, 2018 ▸). The C6—O2 bond of the hydroxyl group [1.371 (2)Å] and those of the acid moiety [O3—C15 = 1.224 (2) and O4—C15 = 1.317 (2) Å] are in the expected ranges. The C5—O1 bond length to the meth­oxy group is 1.372 (2) Å.

## Supra­molecular features   

**Figure 2:**
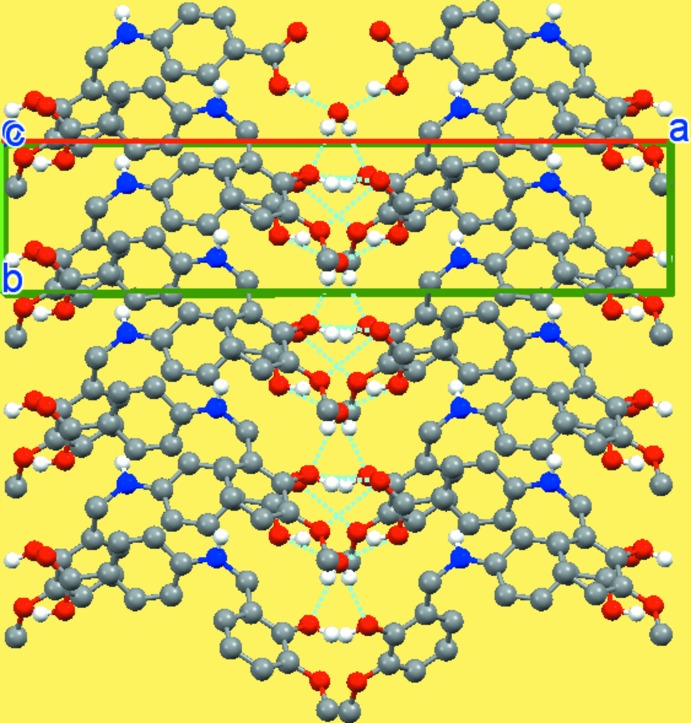
A view of the crystal packing of the title compound.

**Figure 3:**
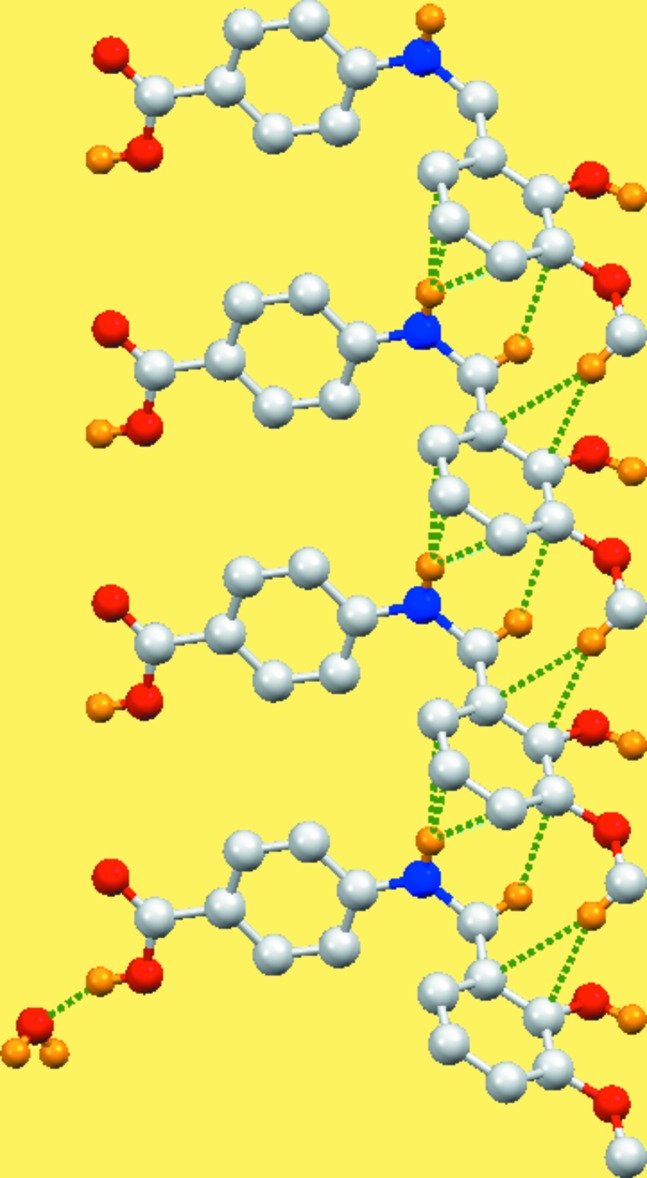
A view along the *c* axis of the zigzag chain in the crystal of the title compound. The N—H⋯*π* inter­actions are shown as dashed lines (see Table 1 ▸ for details).

In the crystal, mol­ecules are connected *via* O—H⋯O inter­actions forming layers in the *ab* plane (Table 1 ▸, Fig. 2 ▸). While the N—H group is not involved in traditional hydrogen-bonding inter­actions, there are inter­molecular N—H⋯*π* inter­actions within the layers (Table 1 ▸, Fig. 3 ▸).

## Database survey   

A search through the Cambridge Structural Database (CSD, Version 5.39, update Aug 2018; Groom *et al.*, 2016 ▸) gave nine hits for the secondary amine. There are only two examples of similar compounds in the literature: ethyl 4-{[(2-hy­droxy­phen­yl)meth­yl]amino}­benzoate, (I) (WEFQEG; Salman *et al.*, 2017 ▸), and ethyl 4-[(3,5-di-*tert*-butyl-2-hy­droxy­benz­yl)amino]benzoate, (II) (VABTAV;. Shakir *et al.*, 2010 ▸). Other related structures based on benzyl­idene-phenyl-amine are reported as *n*-propyl 4-[2-(4,6-di­meth­oxy­pyrimidin-2-yl­oxy)benzyl­amino]­benzoate, (III) (ILAGIL; Wu *et al.*, 2003 ▸), and [4-(2-hy­droxy­benzyl­amino)­benzoato-κ*O*]tri­phenyl­tin(IV), (IV) (WENXAP; Jiang *et al.*, 2006 ▸), There is also one very similar compound, *viz.* ethyl 4-[(2-hy­droxy­benz­yl)amino]­benzoate (Salman *et al.*, 2017 ▸), in which the 3-meth­oxy group in the title compound is replaced by a hydrogen atom and the carb­oxy­lic acid is replaced by an ester. The torsion angle C_ar­yl_—CH_2_—NH—C_ar­yl_ in the title compound [−83.9 (2)°] compares well to those in I (73.68°), II (77.38°) and IV (−87.28°) despite the difference in substituent groups.

## Synthesis and crystallization   

To a hot stirred solution of 4-amino­benzoic acid (PABA) (1.00 g, 7.2 mmol) in methanol (15 ml) was added vanillin (1.11 g, 7.2 mmol). The resultant mixture was then heated under reflux. After an hour, precipitates were formed. The reaction mixture was heated for about a further 30 minutes for the completion of the reaction, which was monitored through TLC. The reaction mixture was cooled to room temperature, filtered and washed with hot methanol. It was then dried in a vacuum to give (*E*)-4-(2-hy­droxy-3-meth­oxy­benzyl­idene­amino)­benzoic acid (1) in 78% yield.

Compound (1) (1.00 g, 3.7 mmol) was dissolved in 25 mL of methanol and reduced by addition of excess sodium borohydride (0.28 g, 7.4 mmol). The solution was stirred until the yellow colour disappeared. Then the solution was diluted with 8–10 times the volume of water and the pH was adjusted to 6 by addition of 12% HCl. The white precipitate was collected and dried in air. Colourless single crystals of the title compound, suitable for X-ray analysis, were obtained by slow evaporation of a methanol solution.

## Refinement   

Crystal data, data collection and structure refinement details are summarized in Table 2 ▸. The N—H and O—H H atoms were located in difference-Fourier maps and freely refined, while the C-bound H atoms were included in calculated positions and treated as riding, with fixed C—H = 0.93 Å, and *U*_iso_(H) = 1.2*U*_eq_(C,N).

## Supplementary Material

Crystal structure: contains datablock(s) I. DOI: 10.1107/S2056989018018455/ld2147sup1.cif

Structure factors: contains datablock(s) I. DOI: 10.1107/S2056989018018455/ld2147Isup2.hkl

Supporting information file. DOI: 10.1107/S2056989018018455/ld2147Isup3.cml

CCDC reference: 1888072

Additional supporting information:  crystallographic information; 3D view; checkCIF report

## Figures and Tables

**Table 1:** Hydrogen-bond geometry (Å, °) *Cg*1 is the centroid of the C2–C7 ring.

*D*—H⋯*A*	*D*—H	H⋯*A*	*D*⋯*A*	*D*—H⋯*A*
O5—H5⋯O2^i^	0.82 (2)	2.06 (2)	2.8640 (19)	164 (3)
O2—H2*A*⋯O3^ii^	0.93 (3)	1.82 (3)	2.6844 (17)	154 (2)
O4—H4*A*⋯O5	0.94 (3)	1.81 (3)	2.6776 (15)	153 (3)
N1—H1⋯*Cg*1^iii^	0.96 (2)	2.40 (2)	3.3008 (18)	157 (2)

Symmetry codes: (i) 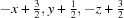; (ii) 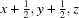; (iii) 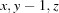.

**Table 2:** Experimental details

Crystal data	
Chemical formula	2C_15_H_15_NO_4_·H_2_O
*M*_r_	564.57
Crystal system, space group	Monoclinic, *C*2/*c*
Temperature (K)	296
*a*, *b*, *c* (Å)	24.742 (3), 5.5002 (6), 19.387 (2)
β (°)	98.292 (6)
*V* (Å^3^)	2610.8 (5)
*Z*	4
Radiation type	Mo *K*α
μ (mm^−1^)	0.11
Crystal size (mm)	0.45 × 0.34 × 0.14

Data collection	
Diffractometer	Bruker APEXII CCD
Absorption correction	Multi-scan (*SADABS*; Bruker, 2014 ▸)
No. of measured, independent and observed [*I* > 2σ(*I*)] reflections	16146, 2570, 2138
*R*_int_	0.065
(sin θ/λ)_max_ (Å^−1^)	0.617

Refinement	
*R*[*F* ^2^ > 2σ(*F* ^2^)], *wR*(*F* ^2^), *S*	0.042, 0.107, 1.05
No. of reflections	2570
No. of parameters	203
No. of restraints	1
H-atom treatment	H atoms treated by a mixture of independent and constrained refinement
Δρ_max_, Δρ_min_ (e Å^−3^)	0.26, −0.22

Computer programs: *APEX2* and *SAINT* (Bruker, 2014 ▸), *SHELXT2014* (Sheldrick, 2015*a* ▸), *SHELXL2018* (Sheldrick, 2015*b* ▸) and *SHELXTL* (Sheldrick, 2008 ▸).

## supplementary crystallographic information

### Crystal data

**Table:**

2C_15_H_15_NO_4_·H_2_O	*F*(000) = 1192
*M**_r_* = 564.57	*D*_x_ = 1.436 Mg m^−^^3^
Monoclinic, *C*2/*c*	Mo *K*α radiation, λ = 0.71073 Å
*a* = 24.742 (3) Å	Cell parameters from 6409 reflections
*b* = 5.5002 (6) Å	θ = 2.5–28.2°
*c* = 19.387 (2) Å	µ = 0.11 mm^−^^1^
β = 98.292 (6)°	*T* = 296 K
*V* = 2610.8 (5) Å^3^	Prism, colorless
*Z* = 4	0.45 × 0.34 × 0.14 mm

### Data collection

**Table:**

Bruker APEXII CCD diffractometer	2138 reflections with *I* > 2σ(*I*)
φ and ω scans	*R*_int_ = 0.065
Absorption correction: multi-scan (SADABS; Bruker, 2014)	θ_max_ = 26.0°, θ_min_ = 2.9°
	*h* = −30→30
16146 measured reflections	*k* = −6→6
2570 independent reflections	*l* = −23→23

### Refinement

**Table:**

Refinement on *F*^2^	1 restraint
Least-squares matrix: full	Hydrogen site location: mixed
*R*[*F*^2^ > 2σ(*F*^2^)] = 0.042	H atoms treated by a mixture of independent and constrained refinement
*wR*(*F*^2^) = 0.107	*w* = 1/[σ^2^(*F*_o_^2^) + (0.0366*P*)^2^ + 3.8398*P*] where *P* = (*F*_o_^2^ + 2*F*_c_^2^)/3
*S* = 1.05	(Δ/σ)_max_ < 0.001
2570 reflections	Δρ_max_ = 0.26 e Å^−^^3^
203 parameters	Δρ_min_ = −0.22 e Å^−^^3^

### Special details

**Table:**

Geometry. All esds (except the esd in the dihedral angle between two l.s. planes) are estimated using the full covariance matrix. The cell esds are taken into account individually in the estimation of esds in distances, angles and torsion angles; correlations between esds in cell parameters are only used when they are defined by crystal symmetry. An approximate (isotropic) treatment of cell esds is used for estimating esds involving l.s. planes.

### Fractional atomic coordinates and isotropic or equivalent isotropic displacement parameters (Å^2^)

**Table:**

	*x*	*y*	*z*	*U*_iso_*/*U*_eq_	
O2	0.95635 (5)	0.7070 (2)	0.67260 (6)	0.0206 (3)	
O5	0.500000	0.7903 (3)	0.750000	0.0222 (4)	
O1	0.97314 (5)	1.0831 (2)	0.59182 (6)	0.0238 (3)	
O3	0.56242 (5)	0.2504 (2)	0.65933 (7)	0.0271 (3)	
O4	0.58989 (5)	0.6076 (3)	0.70737 (8)	0.0347 (4)	
N1	0.81774 (6)	0.2583 (3)	0.63512 (8)	0.0197 (3)	
C14	0.74975 (6)	0.5056 (3)	0.68083 (8)	0.0168 (3)	
H14	0.775566	0.623501	0.696467	0.020*	
C9	0.76511 (6)	0.2985 (3)	0.64627 (8)	0.0158 (3)	
C12	0.65625 (6)	0.3621 (3)	0.66873 (8)	0.0174 (4)	
C15	0.59858 (7)	0.3981 (3)	0.67792 (9)	0.0194 (4)	
C7	0.86973 (6)	0.6259 (3)	0.60621 (9)	0.0177 (4)	
C10	0.72476 (7)	0.1239 (3)	0.62307 (9)	0.0181 (4)	
H10	0.734254	−0.014204	0.599877	0.022*	
C13	0.69600 (7)	0.5340 (3)	0.69160 (8)	0.0174 (3)	
H13	0.686232	0.671711	0.714756	0.021*	
C5	0.92543 (7)	0.9565 (3)	0.57298 (9)	0.0187 (4)	
C6	0.91728 (6)	0.7641 (3)	0.61739 (8)	0.0175 (4)	
C2	0.83105 (7)	0.6781 (3)	0.54820 (9)	0.0206 (4)	
H2	0.799081	0.587223	0.539822	0.025*	
C8	0.86335 (6)	0.4187 (3)	0.65624 (9)	0.0184 (4)	
H8A	0.896629	0.323016	0.662065	0.022*	
H8B	0.859392	0.487346	0.701326	0.022*	
C4	0.88690 (7)	1.0051 (3)	0.51542 (9)	0.0220 (4)	
H4	0.892432	1.131150	0.485232	0.026*	
C11	0.67166 (7)	0.1554 (3)	0.63432 (9)	0.0182 (4)	
H11	0.645659	0.037923	0.618889	0.022*	
C3	0.83997 (7)	0.8641 (3)	0.50316 (9)	0.0236 (4)	
H3	0.814245	0.895045	0.464242	0.028*	
C1	0.98495 (8)	1.2765 (3)	0.54726 (10)	0.0262 (4)	
H1A	0.986748	1.213811	0.501435	0.039*	
H1B	1.019360	1.348797	0.565539	0.039*	
H1C	0.956674	1.397043	0.544826	0.039*	
H5	0.5159 (12)	0.891 (5)	0.7772 (13)	0.076 (10)*	
H1	0.8246 (9)	0.120 (4)	0.6076 (12)	0.038 (6)*	
H2A	0.9900 (10)	0.771 (5)	0.6651 (13)	0.050 (7)*	
H4A	0.5536 (12)	0.630 (6)	0.7145 (15)	0.073 (9)*	

### Atomic displacement parameters (Å^2^)

**Table:**

	*U*^11^	*U*^22^	*U*^33^	*U*^12^	*U*^13^	*U*^23^
O2	0.0122 (6)	0.0235 (7)	0.0262 (6)	−0.0025 (5)	0.0029 (5)	0.0043 (5)
O5	0.0150 (8)	0.0244 (10)	0.0277 (10)	0.000	0.0043 (7)	0.000
O1	0.0199 (6)	0.0212 (7)	0.0313 (7)	−0.0052 (5)	0.0067 (5)	0.0063 (5)
O3	0.0136 (6)	0.0270 (7)	0.0411 (8)	−0.0032 (5)	0.0055 (5)	−0.0011 (6)
O4	0.0173 (7)	0.0366 (8)	0.0524 (9)	0.0000 (6)	0.0127 (6)	−0.0187 (7)
N1	0.0137 (7)	0.0186 (7)	0.0280 (8)	−0.0020 (6)	0.0071 (6)	−0.0034 (6)
C14	0.0150 (8)	0.0163 (8)	0.0191 (8)	−0.0038 (6)	0.0020 (6)	−0.0007 (6)
C9	0.0132 (7)	0.0169 (8)	0.0173 (8)	0.0001 (6)	0.0025 (6)	0.0030 (6)
C12	0.0148 (8)	0.0192 (8)	0.0184 (8)	0.0004 (7)	0.0037 (6)	0.0024 (7)
C15	0.0166 (8)	0.0220 (9)	0.0201 (8)	0.0010 (7)	0.0042 (6)	0.0013 (7)
C7	0.0156 (8)	0.0184 (8)	0.0207 (8)	0.0012 (7)	0.0084 (6)	−0.0023 (7)
C10	0.0176 (8)	0.0145 (8)	0.0224 (8)	0.0004 (7)	0.0035 (7)	−0.0002 (7)
C13	0.0176 (8)	0.0172 (8)	0.0178 (8)	0.0012 (7)	0.0039 (6)	−0.0011 (7)
C5	0.0161 (8)	0.0180 (8)	0.0236 (9)	0.0000 (7)	0.0083 (7)	−0.0010 (7)
C6	0.0149 (8)	0.0187 (8)	0.0198 (8)	0.0030 (7)	0.0057 (6)	−0.0020 (7)
C2	0.0159 (8)	0.0232 (9)	0.0230 (9)	−0.0007 (7)	0.0038 (7)	−0.0033 (7)
C8	0.0116 (7)	0.0196 (9)	0.0246 (9)	−0.0012 (7)	0.0043 (6)	0.0003 (7)
C4	0.0249 (9)	0.0215 (9)	0.0211 (9)	0.0028 (7)	0.0082 (7)	0.0034 (7)
C11	0.0157 (8)	0.0150 (8)	0.0239 (9)	−0.0040 (6)	0.0026 (6)	0.0009 (7)
C3	0.0217 (9)	0.0287 (10)	0.0202 (9)	0.0018 (8)	0.0025 (7)	−0.0001 (7)
C1	0.0290 (10)	0.0180 (9)	0.0351 (10)	−0.0029 (8)	0.0159 (8)	0.0043 (8)

### Geometric parameters (Å, º)

**Table:**

O2—C6	1.371 (2)	C7—C6	1.391 (2)
O2—H2A	0.93 (3)	C7—C2	1.398 (2)
O5—H5	0.823 (17)	C7—C8	1.519 (2)
O5—H5^i^	0.823 (17)	C10—C11	1.374 (2)
O1—C5	1.374 (2)	C10—H10	0.9300
O1—C1	1.427 (2)	C13—H13	0.9300
O3—C15	1.224 (2)	C5—C4	1.385 (2)
O4—C15	1.317 (2)	C5—C6	1.397 (2)
O4—H4A	0.94 (3)	C2—C3	1.383 (3)
N1—C9	1.368 (2)	C2—H2	0.9300
N1—C8	1.445 (2)	C8—H8A	0.9700
N1—H1	0.96 (2)	C8—H8B	0.9700
C14—C13	1.384 (2)	C4—C3	1.388 (3)
C14—C9	1.401 (2)	C4—H4	0.9300
C14—H14	0.9300	C11—H11	0.9300
C9—C10	1.411 (2)	C3—H3	0.9300
C12—C13	1.390 (2)	C1—H1A	0.9600
C12—C11	1.399 (2)	C1—H1B	0.9600
C12—C15	1.477 (2)	C1—H1C	0.9600
			
C6—O2—H2A	109.7 (15)	O1—C5—C6	114.50 (15)
H5—O5—H5^i^	96 (4)	C4—C5—C6	119.92 (16)
C5—O1—C1	117.37 (14)	O2—C6—C7	118.78 (15)
C15—O4—H4A	113.6 (19)	O2—C6—C5	120.42 (15)
C9—N1—C8	125.37 (15)	C7—C6—C5	120.79 (15)
C9—N1—H1	117.7 (13)	C3—C2—C7	120.46 (16)
C8—N1—H1	116.7 (13)	C3—C2—H2	119.8
C13—C14—C9	119.78 (15)	C7—C2—H2	119.8
C13—C14—H14	120.1	N1—C8—C7	115.22 (14)
C9—C14—H14	120.1	N1—C8—H8A	108.5
N1—C9—C14	122.47 (15)	C7—C8—H8A	108.5
N1—C9—C10	119.03 (15)	N1—C8—H8B	108.5
C14—C9—C10	118.50 (14)	C7—C8—H8B	108.5
C13—C12—C11	118.44 (15)	H8A—C8—H8B	107.5
C13—C12—C15	121.46 (15)	C5—C4—C3	119.45 (16)
C11—C12—C15	120.06 (15)	C5—C4—H4	120.3
O3—C15—O4	123.39 (15)	C3—C4—H4	120.3
O3—C15—C12	123.62 (16)	C10—C11—C12	120.71 (15)
O4—C15—C12	112.98 (15)	C10—C11—H11	119.6
C6—C7—C2	118.60 (16)	C12—C11—H11	119.6
C6—C7—C8	118.28 (15)	C2—C3—C4	120.72 (16)
C2—C7—C8	123.08 (15)	C2—C3—H3	119.6
C11—C10—C9	120.84 (15)	C4—C3—H3	119.6
C11—C10—H10	119.6	O1—C1—H1A	109.5
C9—C10—H10	119.6	O1—C1—H1B	109.5
C14—C13—C12	121.73 (15)	H1A—C1—H1B	109.5
C14—C13—H13	119.1	O1—C1—H1C	109.5
C12—C13—H13	119.1	H1A—C1—H1C	109.5
O1—C5—C4	125.57 (16)	H1B—C1—H1C	109.5
			
C8—N1—C9—C14	−0.4 (3)	C8—C7—C6—C5	179.87 (15)
C8—N1—C9—C10	−179.51 (15)	O1—C5—C6—O2	−3.0 (2)
C13—C14—C9—N1	−178.88 (15)	C4—C5—C6—O2	176.85 (15)
C13—C14—C9—C10	0.2 (2)	O1—C5—C6—C7	177.53 (14)
C13—C12—C15—O3	−179.11 (16)	C4—C5—C6—C7	−2.7 (2)
C11—C12—C15—O3	3.0 (3)	C6—C7—C2—C3	−0.2 (2)
C13—C12—C15—O4	1.8 (2)	C8—C7—C2—C3	−177.81 (16)
C11—C12—C15—O4	−176.06 (15)	C9—N1—C8—C7	−83.9 (2)
N1—C9—C10—C11	178.86 (15)	C6—C7—C8—N1	−170.01 (14)
C14—C9—C10—C11	−0.3 (2)	C2—C7—C8—N1	7.6 (2)
C9—C14—C13—C12	−0.3 (2)	O1—C5—C4—C3	−179.06 (16)
C11—C12—C13—C14	0.3 (2)	C6—C5—C4—C3	1.2 (2)
C15—C12—C13—C14	−177.56 (15)	C9—C10—C11—C12	0.4 (3)
C1—O1—C5—C4	−2.0 (2)	C13—C12—C11—C10	−0.4 (2)
C1—O1—C5—C6	177.78 (14)	C15—C12—C11—C10	177.54 (15)
C2—C7—C6—O2	−177.34 (14)	C7—C2—C3—C4	−1.3 (3)
C8—C7—C6—O2	0.3 (2)	C5—C4—C3—C2	0.8 (3)
C2—C7—C6—C5	2.2 (2)		

Symmetry code: (i) −*x*+1, *y*, −*z*+3/2.

### Hydrogen-bond geometry (Å, º)

*Cg*1 is the centroid of the C2–C7 ring.

**Table:**

*D*—H···*A*	*D*—H	H···*A*	*D*···*A*	*D*—H···*A*
O5—H5···O2^ii^	0.82 (2)	2.06 (2)	2.8640 (19)	164 (3)
O2—H2*A*···O3^iii^	0.93 (3)	1.82 (3)	2.6844 (17)	154 (2)
O4—H4*A*···O5	0.94 (3)	1.81 (3)	2.6776 (15)	153 (3)
N1—H1···*Cg*1^iv^	0.96 (2)	2.40 (2)	3.3008 (18)	157 (2)

Symmetry codes: (ii) −*x*+3/2, *y*+1/2, −*z*+3/2; (iii) *x*+1/2, *y*+1/2, *z*; (iv) *x*, *y*−1, *z*.
